# Predicting insulin resistance in non-diabetics: Comparing obesity indicators using machine learning

**DOI:** 10.1097/MD.0000000000051034

**Published:** 2026-10-16

**Authors:** Zhiyu Guo, Yingfeng Wei, Xuechao Lu, Chunhua Wang

**Affiliations:** a Division of Endocrinology and Metabolism, Department of Internal Medicine, Nantong Third People’s Hospital, Nantong University, Nantong, Jiangsu, China.

**Keywords:** insulin resistance, machine learning, NHANES, obesity indicators

## Abstract

This cross-sectional study aimed to compare obesity indicators for predicting insulin resistance using machine learning. Data were obtained from NHANES 2015 to 2018, focusing on a nondiabetic adult population. Insulin resistance was defined as HOMA-IR ≥ 75th percentile of the training set. Key predictors were selected via bootstrap stability selection with elastic net. Logistic regression, random forest, and extreme gradient boosting (XGBoost) models were built using body mass index (BMI) and waist-to-height ratio (WHtR). Performance was assessed by area under the curve (AUC), sensitivity, specificity, accuracy, calibration, and decision curve analysis. Race-stratified and nested regression analyses were performed. A total of 3562 subjects were included. BMI, WHtR, Glycated hemoglobin, and triglycerides were identified as key predictors. All models achieved AUC > 0.79; logistic regression (LR) performed best (BMI-based LR: AUC = 0.811; WHtR-based LR: AUC = 0.811). WHtR-based LR showed slightly better accuracy (0.746 vs 0.715) and specificity (0.744 vs 0.678). Obesity indicators had the highest feature importance, and race-stratified analyses confirmed general robustness across subgroups. WHtR and BMI are strong independent predictors of IR. LR models using these simple indices performed well in discrimination, calibration, and net benefit, offering cost-effective screening options, particularly where resources are scarce. Future work should prioritize external validation and race-specific thresholds.

## 1. Introduction

In recent years, the global prevalence of overweight and obesity has continued to rise. According to World Health Organization (WHO) data, the global prevalence of obesity among adults increased from 7% in 1990 to 16% in 2022. In adults, overweight is defined as a body mass index (BMI) ≥ 25 kg/m^2^, while obesity is defined as a BMI ≥ 30 kg/m^2^.^[1]^ Obesity significantly increases the risk of numerous noninfectious diseases, including type 2 diabetes mellitus (T2DM), cardiovascular diseases, certain cancers, neurological disorders, and chronic respiratory conditions, making it one of the most critical public health challenges worldwide.^[2]^

The pathophysiological basis of obesity lies in the excessive accumulation of body fat. Abnormal fat distribution, especially visceral or abdominal adiposity (also referred to as central obesity), plays a central role in the development of insulin resistance (IR).^[3,4]^ Commonly used anthropometric indices for obesity assessment, such as BMI, waist circumference, waist-to-height ratio (WHtR), and hip circumference, provide a simple, cost-effective, clinically practical, and highly reproducible means of evaluating adiposity. Compared with BMI, waist circumference and WHtR more accurately reflect abdominal fat accumulation and have been shown to be stronger predictors of central obesity and IR.^[5,6]^

Machine learning, which enables computer systems to automatically learn patterns from data and make intelligent decisions through algorithmic training, has become an essential analytical tool in public health research.^[7]^ IR, as a common pathological basis for metabolic diseases such as type 2 diabetes mellitus and cardiovascular diseases, holds significant clinical importance for early identification and intervention. Currently, the HOMA-IR is the primary method for evaluating IR in clinical practice. This index is derived from fasting insulin and fasting blood glucose levels. However, since insulin assay is not always included in routine testing, the widespread application of HOMA-IR remains somewhat limited. Consequently, numerous studies have attempted to use machine learning algorithms to predict IR in nondiabetic populations.

However, these existing studies predominantly rely on BMI as the primary obesity evaluation criterion.^[8,9]^ This study introduces and compares multiple anthropometric indices for obesity assessment, such as BMI, WHtR and weight-adjusted waist index (WWI), to identify the parameters most strongly associated with IR.

## 2. Materials and methods

### 2.1. Data source

Data were obtained from the National Health and Nutrition Examination Survey (NHANES), which collects comprehensive health-related data through physical examinations, laboratory tests, and dietary interviews. The survey employs a stratified, multistage probability sampling design to ensure national representativeness. This study included adult participants (aged ≥20 years) from the 2015 to 2018 NHANES cycles.

### 2.2. Study population

From an initial cohort of 19,225 people, 5454 individuals with fasting blood samples were selected for analysis. Participants who had been diagnosed with diabetes, or who met the WHO diagnostic criteria (WHO, 2011) for fasting plasma glucose (≥7.0 mmol/L) or glycated hemoglobin (HbA1c) (≥6.5%), were excluded. Individuals under 20 years of age were also removed. A total of 3562 participants were included in the final analysis, as shown in Figure 1.

**Figure 1.**
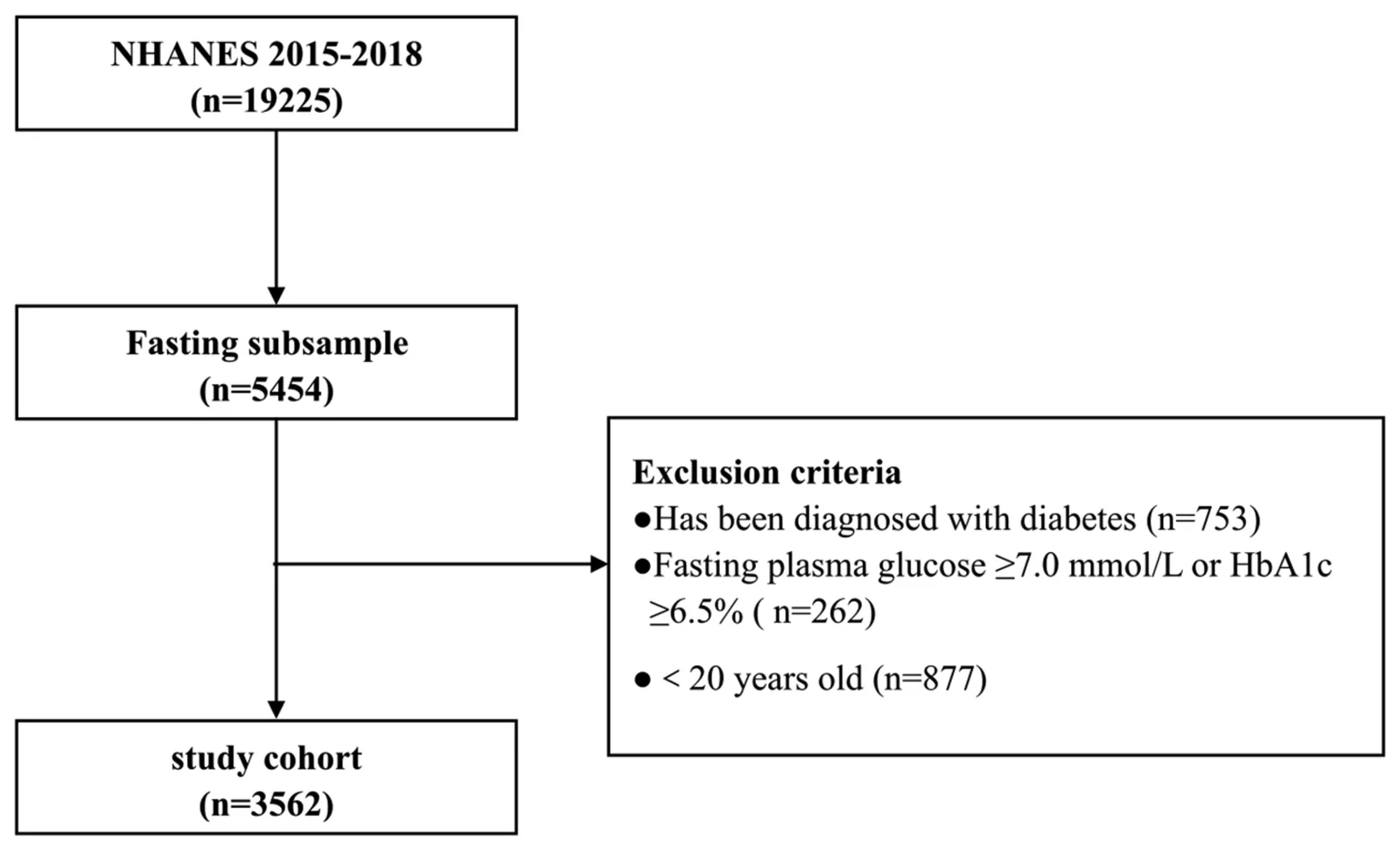
Flow Chart of Participant Selection. HbA1c = glycated hemoglobin, NHANES = National Health and Nutrition Examination Survey.

Diabetes was defined by: Self‑reported physician diagnosis: affirmative responses to “Did a doctor tell you that you have diabetes?,” “Are you taking insulin now?,” or “Are you taking diabetes pills to lower your blood sugar?.”

### 2.3. Variables

The following data were extracted: demographic characteristics (such as gender, age, race), anthropometric measurements (such as height, weight, waist circumference), and clinical parameters including systolic and diastolic blood pressure, C-reactive protein, total cholesterol, triglycerides (TG), low-density lipoprotein cholesterol, fasting plasma glucose, fasting insulin, and HbA1c.

The following obesity indices were calculated:

- BMI: weight (kg)/ height^2^ (m^2^).
- WHtR: waist circumference (cm)/ height (cm).
- WWI: waist circumference (cm)/ √weight (kg).

IR status was assessed using HOMA-IR, calculated as:


HOMA-IR=[Fasting Insulin(pmol/L)×Fasting Glucose(mmol/L)]/ 22.5.

### 2.4. Variable selection and model building

The included data were randomly split into a training set (70%) and a test set (30%).

The cutoff value for HOMA-IR was defined as the 75th percentile of its distribution in the training set.^[10]^ Participants with HOMA-IR values at or above this cutoff were classified as having high IR, whereas those below it were classified as the low IR group.

In the training set, all continuous variables were standardized using z-score normalization before analysis. We used bootstrap stability selection with elastic net penalized logistic regression (LR) to identify useful predictors of IR. We generated 100 bootstrap resamples from the original training set. In each bootstrap sample, we fit an LR model with an elastic net penalty, setting α to 0.5 to balance the L1 and L2 penalties equally. We used 10-fold cross-validation to select the penalty parameter λ by the one-standard-error rule (λ.1se), based on the cross-validated area under the curve (AUC). Predictors with nonzero coefficients in more than 50% of the bootstrap replicates were considered stable and retained for further analysis.

Although race was not selected as a predictor during the feature selection procedure, it was forced into all models as a covariate to adjust for potential confounding, given the racial differences in body composition and adiposity distribution. We also performed stratification analyses by race on the final selected model to verify the robustness of the model performance across racial subgroups.

Three machine learning algorithms, namely LR, random forest (RF), and extreme gradient boosting (XGBoost), were employed to develop prediction models for IR status using BMI and WHtR, respectively. For RF and XGBoost, hyperparameter tuning was performed using 5-fold cross-validation stratified by the outcome variable. The hyperparameters that gave the best mean AUC across the 5 folds were selected. A fixed random seed (123) was applied throughout to ensure reproducibility.

For RF, the grid search covered mtry ∈ {2, 3, 4} and min.node.size ∈ {5, 10, 20}, yielding 9 candidate combinations; the optimal one was selected based on the highest mean cross-validated AUC (final: mtry = 4, min.node.size = 20).

For XGBoost, 3 candidate parameter sets were evaluated: (a) max_depth = 3, gamma = 0.1, subsample = 0.7, colsample_bytree = 0.7, min_child_weight = 5; (b) max_depth = 4, gamma = 0, subsample = 0.8, colsample_bytree = 0.8, min_child_weight = 3; and (c) max_depth = 3, gamma = 0.2, subsample = 0.6, colsample_bytree = 0.6, min_child_weight = 10. For each set, 5-fold cross-validation with early stopping (patience = 20 rounds, max 1000 rounds) determined nrounds. The best-performing set (highest mean AUC) was selected (final: max_depth = 4, gamma = 0, subsample = 0.8, colsample_bytree = 0.8, min_child_weight = 3, nrounds = 17).

After hyperparameter optimization, the final models were refit using the full training set with the optimal hyperparameters and evaluated on the test set.

### 2.5. Model evaluation

Model performance on the test set was assessed using ROC-AUC, sensitivity, and specificity. Differences in AUC between models were compared using DeLong’s test. For the final model, calibration and clinical utility were further evaluated using a calibration plot and decision curve analysis.

### 2.6. Variable importance assessment

To quantify the incremental value of anthropometric indices beyond metabolic parameters, we built a series of nested LR models. Model 1 included metabolic parameters and race. Model 2 included WHtR and race. Model 3 included BMI and race. Model 4 added WHtR to Model 1. Model 5 added BMI to Model 1. Incremental predictive performance was assessed by ΔAUC with 95% CI, tested via DeLong’s method. ORs with 95% CIs were also computed for predictors.

### 2.7. Statistical analysis

All statistical analyses were performed using R software (version 4.4.3). Given NHANES’s complex, multistage probability sampling design and the use of the fasting subsample, all analyses incorporated the recommended survey weights (WTSAF2YR), strata (SDMVSTRA), and primary sampling units (SDMVPSU), following NCHS guidelines. Outliers were identified by the interquartile range (IQR) method (values below Q1 −1.5 × IQR or above Q3 + 1.5 × IQR) and winsorized. Missing data were imputed using multiple imputation by chained equations, with predictive mean matching for continuous variables and LR for categorical variables.

Continuous variables with an approximately normal distribution were presented as mean ± standard deviation (SD), with group comparisons performed using survey-weighted *t* tests. Non-normally distributed continuous variables were presented as median with IQR, with group comparisons performed using survey-weighted rank-sum tests. Categorical variables were presented as frequencies (percentages), with group comparisons performed using Rao–Scott chi-square tests accounting for survey weights. A two-sided *P*-value <.05 was considered statistically significant.

## 3. Results

### 3.1. Baseline characteristics

A total of 3562 participants were included in the final analysis, as shown in Figure 1. The baseline characteristics of the training and test sets are summarized in Table 1. No significant differences were observed between the 2 sets across all demographic, anthropometric, and metabolic variables (all *P* > .05).

**Table 1:** Baseline characteristics.

Characteristic	Training Set	Test Set	*P*
	n (2493)	n (1069)	
Gender, n (%)			.714
Male	1161 (46.6)	499 (46.7)	
Female	1326 (53.2)	557 (52.1)	
Age, yr	46.06 (16.67)	45.59 (17.38)	.479
Race, n (%)			.973
Mexican American	365 (14.6%)	151 (14.1%)	
Other Hispanic	279 (11.2%)	124 (11.6%)	
Non-Hispanic White	863 (34.6%)	382 (35.7%)	
Non-Hispanic Black	532 (21.3%)	223 (20.9%)	
Other	454 (18.2%)	189 (17.7%)	
Height, cm	168.10 (9.58)	168.48 (9.96)	.442
Weight, kg	78.20 (66.77, 95.30)	79.48 (67.11, 92.50)	.823
Waist, cm	98.92 (16.23)	98.09 (15.37)	.349
BMI, kg/m^2^	27.80 (23.88, 33.00)	27.80 (24.32, 31.70)	.427
WHtR	0.59 (0.10)	0.58 (0.10)	.319
WWI	10.97 (0.85)	10.93 (0.87)	.361
SBP, mm Hg	121.42 (16.41)	121.77 (16.60)	.625
DBP, mm Hg	70.68 (11.18)	70.63 (11.13)	.924
FPG, mmol/L	5.60 (0.53)	5.62 (0.52)	.513
FINS, pmol/L	51.66 (34.08, 83.52)	49.54 (32.16, 83.94)	.492
HOMA-IR	1.86 (1.17, 3.04)	1.75 (1.14, 3.08)	.487
HbA1c, %	5.42 (0.35)	5.40 (0.36)	.328
TC, mmol/L )	4.93 (0.99)	4.93 (1.03)	.921
TG, mmol/L	0.99 (0.66, 1.47)	0.96 (0.67, 1.45)	.510
LDL-C, mmol/L	2.93 (0.86)	2.93 (0.89)	.858
CRP, mg/L	1.69 (0.73, 3.87)	1.60 (0.67, 3.90)	.484

Continuous variables are presented as mean (SD) for normally distributed variables or median (IQR) for non‑normally distributed variables; categorical variables are presented as n (weighted %). *P* values are derived from survey‑weighted *t* tests, rank‑sum tests, or Rao‑Scott chi‑square tests, as appropriate.

CRP = C-reactive protein, DBP = diastolic blood pressure, FINS = fasting insulin, FPG = fasting plasma glucose, HbA1c = glycated hemoglobin, LDL-C-density lipoprotein cholesterol, SBP = systolic blood pressure, TC = total cholesterol, TG = triglycerides, WHtR = waist-to-height ratio, WWI = weight-adjusted waist index.

### 3.2. Preliminary comparison of obesity indicators and feature screening

As shown in Figure 2A, WHtR, TG, BMI, and HbA1c were selected in more than 50% of the bootstrap replicates and were considered stable predictors. WHtR, TG, and BMI had selection frequencies close to 100%, whereas HbA1c was selected in about 80%. Diastolic blood pressure, Age, total cholesterol, C-reactive protein, Race, Gender, LDL, WWI, and Systolic Blood Pressure fell below 50% and were not retained. The selection frequency heatmap further supported these findings (Fig. 2C). All 4 stable predictors had positive effects on the outcome, with BMI showing the largest regression coefficient, followed by WHtR, TG, and HbA1c (Fig. 2B).

**Figure 2.**
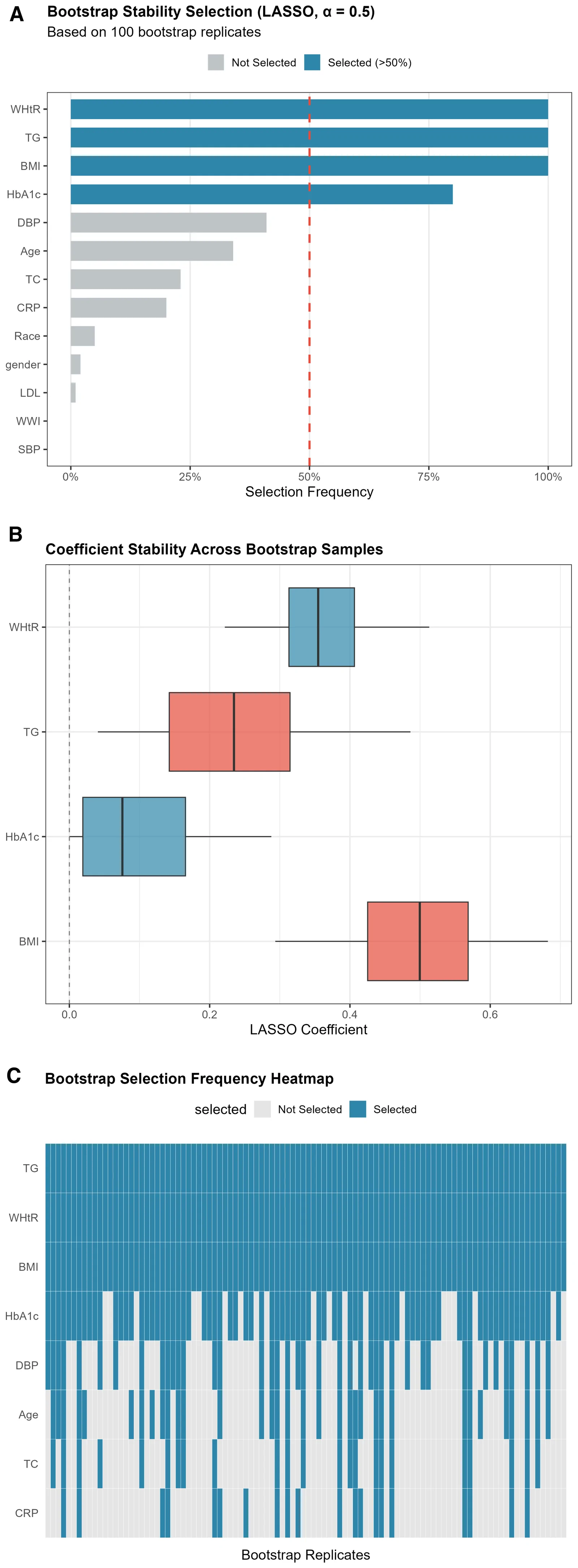
Bootstrap stability selection analysis of variables based on elastic-net LASSO regression. (A) Variable selection frequency over 100 bootstrap resamples. The red dashed line marks the 50% stability threshold; variables above this line were deemed robust predictors. (B) Bootstrap distributions of regression coefficients for stable variables, all of which exhibited positive coefficients, indicating a risk-enhancing effect. (C) Selection status heatmap across bootstrap replicates, with blue indicating variable inclusion and gray indicating exclusion.

### 3.3. Three machine learning model performance comparison

Table 2 summarizes the performance of the LR, RF, and XGBoost models based on BMI and WHtR. The corresponding ROC curves are shown in Figure 3A (BMI-based) and Figure 3B (WHtR-based), with a direct comparison of the 2 optimal LR models in Figure 3C. AUCs ranged from 0.795 to 0.811 across all models, indicating good discriminative ability. BMI-based LR and WHtR-based LR achieved the highest AUCs (both 0.811; *P* > .05). BMI-based LR showed higher sensitivity (0.824), whereas WHtR-based LR had a more balanced profile (sensitivity 0.753, specificity 0.744, accuracy 0.746). Overall, LR models outperformed RF and XGBoost models.

**Table 2:** Predictive performance of models for insulin resistance.

Model	AUC (95% CI)	Cutoff	Sensitivity	Specificity	Accuracy
BMI-based LR	0.811 (0.783–0.840)	0.177	0.824	0.678	0.715
WHtR-based LR	0.811 (0.783–0.840)	0.248	0.753	0.744	0.746
BMI-based XGBoost	0.805 (0.775–0.835)	0.349	0.693	0.783	0.761
WHtR-based XGBoost	0.808 (0.779–0.836)	0.304	0.727	0.739	0.736
BMI-based RF	0.797 (0.767–0.827)	0.172	0.843	0.603	0.663
WHtR-based RF	0.795 (0.766–0.825)	0.265	0.790	0.700	0.722

Cutoff values are determined by maximizing the Youden index. Sensitivity, specificity, and accuracy are calculated at the corresponding cutoff points. All models are evaluated on the test set.

CI = confidence interval, LR = logistic regression, RF = random forest, WHtR = waist-to-height ratio, XGBoost = extreme gradient boosting.

**Figure 3.**
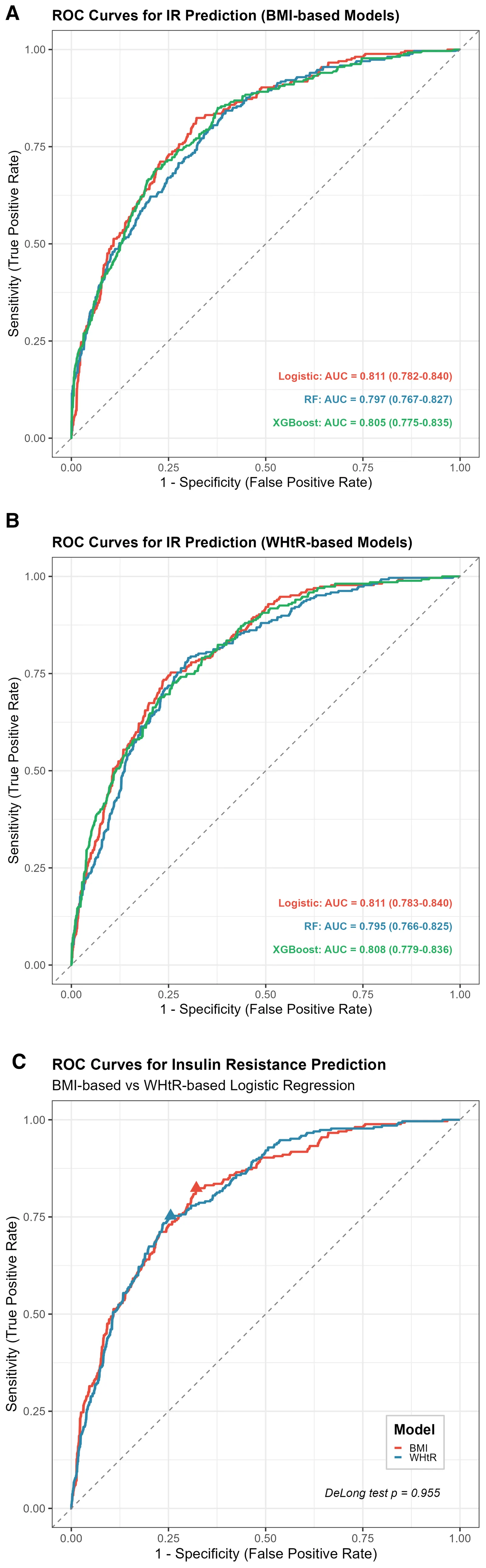
Receiver operating characteristic curves for prediction models of insulin resistance. (A) BMI‑based logistic regression, random forest, and XGBoost models. (B) WHtR‑based logistic regression, random forest, and XGBoost models. (C) Comparison of the 2 logistic regression models. AUC values with 95% CIs are shown. The DeLong test for the comparison between BMI‑based and WHtR‑based logistic regression models yielded *P* = .955, indicating no statistically significant difference. AUC = area under the curve, BMI = body mass index, WHtR = Waist-to-Height Ratio.

### 3.4. Calibration and decision curve analysis of the LR models

Calibration curves for the BMI-based LR and WHtR-based LR models are shown in Figure 4A. Both curves lay close to the ideal 45-degree reference line, indicating satisfactory calibration. Predicted risks of IR were generally consistent with observed incidences, with only minor deviations in the high-risk probability region.

**Figure 4.**
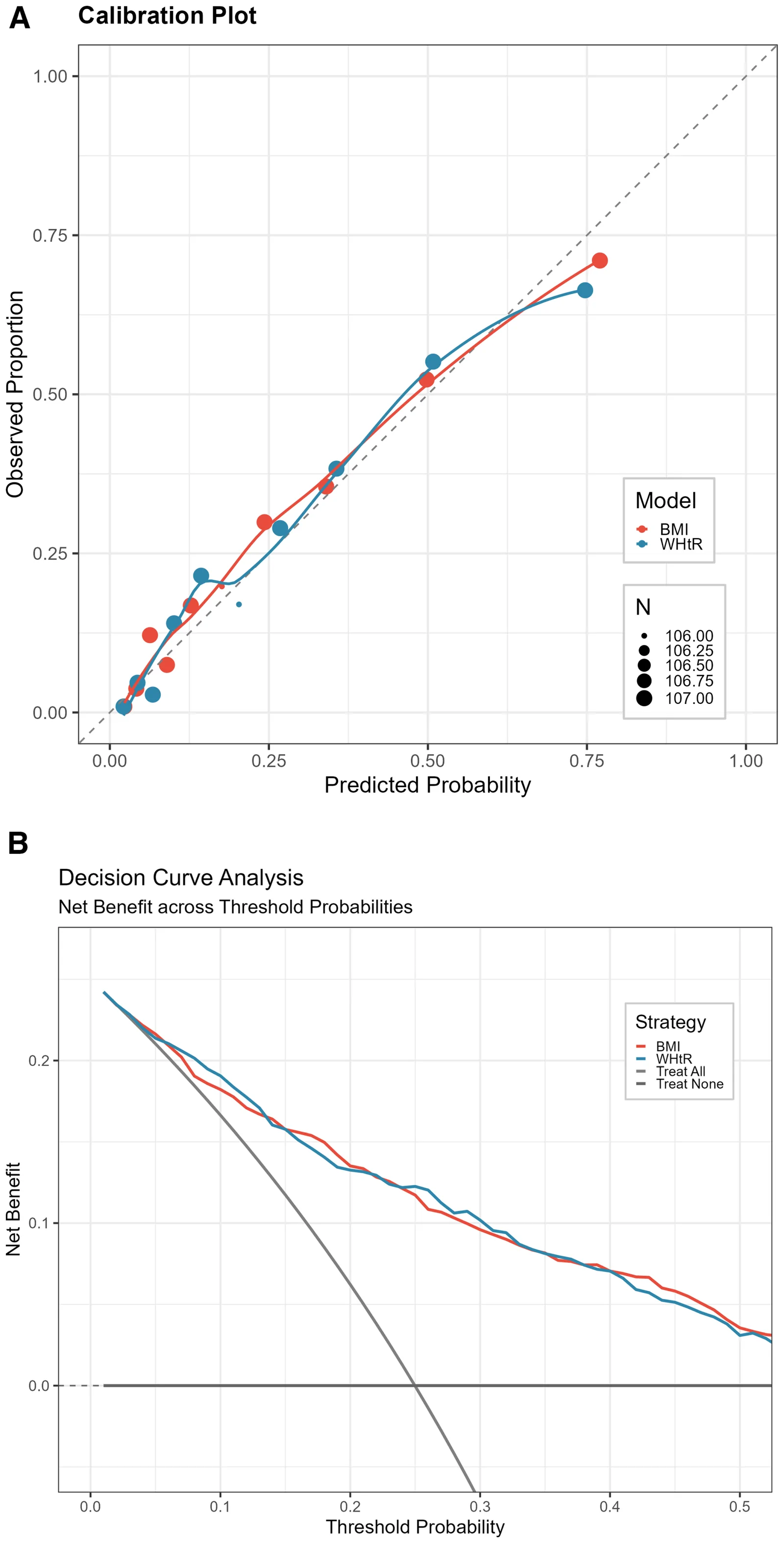
Calibration plot and decision curve analysis for BMI-based and WHtR-based logistic regression models. (A) Calibration plot. The x-axis represents the predicted probability, and the y-axis indicates the observed proportion. The dashed diagonal line denotes the ideal reference for perfect calibration; point size reflects the sample size per risk decile. (B) Decision curve analysis. The x-axis shows the threshold probability, and the y-axis presents the net benefit. The “Treat All” and “Treat None” lines represent intervening in all patients and in none, respectively. Both BMI- and WHtR-based models demonstrated positive net benefit across a wide range of threshold probabilities. BMI = body mass index, WHtR = Waist-to-Height Ratio.

Decision curve analysis is shown in Figure 4B. Within the threshold probability range of 0 to 0.55, both models yielded positive net benefits and outperformed the “treat all” and “treat none” strategies. The WHtR-based LR showed a slightly higher net benefit than the BMI-based LR, although their overall clinical utility was comparable. (Fig. 4B

### 3.5. Stratified analysis of the WHtR-based LR model by race

To evaluate whether the performance of the final LR model varied across racial subgroups, we conducted stratification analyses by race. As presented in Table 3, the model’s AUC ranged from 0.725 (Other Hispanic) to 0.853 (Non-Hispanic White and Other). The highest accuracy was observed in the Other race group (0.825), whereas the lowest was in the Mexican American group (0.642). Sensitivity ranged from 0.596 (Non-Hispanic Black) to 0.771 (Mexican American), and specificity from 0.583 (Mexican American) to 0.862 (Other). Collectively, these findings demonstrate that the model exhibits generally consistent discriminatory performance across racial subgroups, although predictive accuracy appeared somewhat diminished among Other Hispanic and Mexican American populations.

**Table 3:** Predictive performance of the WHtR-based logistic regression model across racial subgroups.

Race	AUC	Sensitivity	Specificity	Accuracy
Mexican American	0.763	0.771	0.583	0.642
Other Hispanic	0.725	0.750	0.652	0.677
Non-Hispanic White	0.853	0.729	0.811	0.791
Non-Hispanic Black	0.766	0.596	0.790	0.749
Other	0.853	0.705	0.862	0.825

Performance metrics are evaluated on the test set. AUC values ranged from 0.725 to 0.853, suggesting generally consistent discriminatory performance across racial subgroups.

WHtR=waist‑to‑height ratio.

### 3.6. Incremental predictive value assessed by nested regression

The nested LR models are summarized in Table 4. The baseline model (TG + HbA1c + Race) had an AUC of 0.724. Adding WHtR or BMI significantly improved the AUC to 0.811 (ΔAUC = 0.087; both *P* < .001). In the full models, WHtR (OR = 3.75) and BMI (OR = 4.02) remained strong independent predictors of IR, while the ORs of TG and HbA1c were attenuated but remained significant.

**Table 4:** Nested logistic regression models: incremental value of WHtR and BMI over metabolic parameters for predicting insulin resistance.

Model	AUC (95% CI)	Key predictors, OR (95% CI)	*P* (vs Model1)
Model1: Metabolic + Race	0.724 (0.690–0.758)	TG: 1.60 (1.41–1.83) HbA1c: 1.58 (1.29–1.94)	–
Model2: WHtR + Race	0.786 (0.757–0.816)	WHtR: 4.04 (3.25–5.02)	<.001
Model3: BMI + Race	0.783 (0.752–0.813)	BMI: 4.25 (3.28–5.51)	<.001
Model4: WHtR + Metabolic + Race	0.811 (0.783–0.840)	WHtR: 3.75 (3.00–4.68) TG: 1.48 (1.28–1.72) HbA1c: 1.26 (1.01–1.57)	<.001
Model5: BMI + Metabolic + Race	0.811 (0.782–0.840)	BMI: 4.02 (3.10–5.21) TG: 1.48 (1.27–1.73) HbA1c: 1.42 (1.13–1.80)	<.001

Metabolic parameters include TG and HbA1c. All models are adjusted for race. AUCs are calculated on the test set. ORs represent the change in odds per 1‑standard‑deviation increase in the predictor.

CI = confidence interval, HbA1c = glycated hemoglobin, OR = odds ratio, TG = triglycerides, WHtR = waist‑to‑height ratio.

## 4. Discussion

In this study, we developed and validated LR, XGBoost, and RF models using WHtR and BMI based on NHANES data to predict IR. Our results demonstrate that both WHtR and BMI are strong independent predictors of IR. The final LR model exhibited good discrimination (AUC = 0.811), satisfactory calibration, and positive net benefit across clinically relevant threshold probabilities. Stratified analyses by race showed consistent model performance across subgroups, further supporting the robustness of our findings.

Both WHtR (OR = 3.75) and BMI (OR = 4.02) demonstrated strong independent associations with IR. Although previous studies have reported that WHtR may be superior to BMI in predicting IR,^[11–16]^ we did not observe a significant difference between the 2 indices. This discrepancy may be attributable to the predominantly U.S. composition of our study population, where general obesity is more prevalent, and the “lean IR” phenotype is less commonly encountered.^[17,18]^ Our findings are consistent with the concept of obesity heterogeneity proposed by Blüher, suggesting that the relative contributions of general and central adiposity to metabolic risk may differ across populations.^[19]^

Regardless of whether the models were based on WHtR or BMI, LR models consistently achieved the highest AUC on the test set, outperforming XGBoost and RF (WHtR-based: 0.811 vs 0.808 vs 0.795; BMI-based: 0.811 vs 0.805 vs 0.797). This finding is consistent with previous studies,^[20,21]^ suggesting that LR is comparable to or better than more complex machine learning algorithms for anthropometric variables. More complex machine learning methods only outperform LR when the data are high-dimensional and biochemical. Thus, LR remains the preferred choice when using anthropometric indices to screen for IR, offering a favorable balance of accuracy, interpretability, and clinical utility.

The racial variations observed in our model performance are consistent with previous findings. Cho et al reported that the AUC of the SPISE index for predicting metabolic syndrome varied significantly by race, with the highest performance in White and the lowest in Black populations.^[22]^ Our finding that the model performed best in Non-Hispanic White (AUC = 0.853) and least well in Hispanic (AUC = 0.725) aligns with the well-documented observation that Hispanic populations have higher susceptibility to IR and metabolic dysfunction. These disparities likely reflect underlying differences in body composition, fat distribution, genetic susceptibility, and the relationship between adiposity and HOMA-IR across racial groups.

This study has several limitations. First, the cross-sectional design of NHANES precludes causal inference. Second, IR was defined using HOMA-IR rather than the hyperinsulinemic-euglycemic clamp technique, which remains the gold standard. Third, the use of the fasting subsample may limit the representativeness of our study population, as individuals with complete fasting data may differ systematically from the overall NHANES sample. Finally, our findings are based on the U.S. population and may not be directly generalizable to other settings without external validation.

## 5. Conclusions

In conclusion, WHtR and BMI are strong independent predictors of IR, with significant incremental predictive value over metabolic parameters. The LR models based on these simple anthropometric indices demonstrated good discrimination, satisfactory calibration, and positive net benefit, supporting their use as practical, cost-effective screening tools for IR, particularly in resource-limited settings. Future research should focus on external validation in diverse populations and prospective cohorts, as well as the development of race-specific cutoff values to further optimize clinical utility.

## Author contributions

**Formal analysis:** Zhiyu Guo.

**Funding acquisition:** Zhiyu Guo.

**Supervision:** Yingfeng Wei.

**Data curation:** Xuechao Lu.

**Methodology:** Chunhua Wang.

**Writing – original draft:** Zhiyu Guo.

**Writing – review & editing:** Yingfeng Wei, Chunhua Wang.
